# AI-assisted age estimation from occlusal tooth wear using biofluorescence imaging

**DOI:** 10.1038/s41598-026-42573-1

**Published:** 2026-04-02

**Authors:** Sang-Kyeom Kim, Eun-Song Lee, Baek-Il Kim

**Affiliations:** Department of Preventive Dentistry & Public Oral Health, BK21 FOUR Project, Yonsei University College of Dentistry, Seoul, Korea

**Keywords:** Forensic odontology, Quantitative light-induced fluorescence, Random forest, Feature selection, Explainable artificial intelligence, Computational biology and bioinformatics, Diseases, Health care, Medical research

## Abstract

This proof-of-concept study evaluated the feasibility of an AI-based age estimation model using an occlusal tooth wear parameter (ΔF_wear_) quantified from biofluorescence. Quantitative light-induced fluorescence (QLF) images from 104 adults (20–70 years; 2,733 teeth) were analyzed. To prevent data leakage, the dataset was split at the participant level. A random forest (RF) regressor was optimized, and recursive feature elimination with cross-validation (RFECV) identified efficient tooth subsets. Final models were validated using an independent test set, and correlations between mean ΔF_wear_ and chronological age were assessed. Cross-validation (CV) performance peaked with three teeth; however, independent testing showed that a model incorporating seven key teeth achieved the best generalization performance. This 7-tooth model achieved a mean absolute error (MAE) of 7.49 years (95% CI: 5.90–9.17), comparable to the full 28-tooth model (MAE: 7.27 years; *p* = 0.79), with a stronger Pearson correlation with age (*r* = 0.78 vs. 0.71) and an equivalent R^2^ of 0.61. These findings support the feasibility of integrating ΔF_wear_ with an interpretable machine-learning framework for non-invasive age estimation. While the reduced 7-tooth model offers analytical efficiency, further validation in larger and more diverse cohorts is required to confirm its generalizability for broader forensic or epidemiological applications.

## Introduction

Accurate age estimation using dental characteristics is crucial in various fields, including forensic science and clinical dentistry^1,2^. As a highly mineralized tissue, teeth are exceptionally durable against environmental insults and are well-preserved post-mortem, making them a reliable indicator for determining age^3,4^. Traditionally, various methods have been employed for adult age estimation, such as dental radiography^5,6^, histological analysis^7,8^, and morphometric assessments^9^. Among these, the degree of occlusal tooth wear or attrition has long been recognized as a cumulative indicator of aging. Consequently, forensic dentistry has often relied on grading systems for occlusal wear to evaluate its correlation with age^10^. However, these visual assessment methods depend on ambiguous descriptive criteria and have been criticized for their significant variability, which is contingent on the examiner’s experience and calibration standards^11,12^. For instance, Ganss et al. reported that the visual detection of dentin exposure on worn teeth yielded only ‘fair’ inter-examiner agreement (κ = 0.28) and ‘moderate’ intra-examiner agreement (κ = 0.55), highlighting the limitations of subjective evaluations^13^.

Quantitative light-induced fluorescence (QLF), a biofluorescence technology, was originally developed for the detection of initial dental caries^14^. This optical method involves irradiating the tooth with 405 nm blue light and quantifying the resulting biofluorescence at wavelengths above 520 nm. While sound enamel exhibits intrinsic fluorescence, demineralized areas in early caries show a decrease in fluorescence intensity, enabling an early diagnosis^15^. Recently, research has extended the application of QLF to the assessment of tooth wear, capitalizing on the altered fluorescence characteristics of worn enamel and exposed dentin. Analyses of fluorescence images of worn teeth revealed that the worn areas exhibited higher fluorescence intensity than the surrounding sound enamel. A quantified metric of this difference, ΔF_wear_, reflects the severity of wear^16^. Lee et al.^17^ reported that the difference in fluorescence intensity (ΔG) measured by QLF using extracted teeth showed a significant correlation with the conventional Tooth Wear Index (TWI) of Smith and Knight^18^ and could significantly distinguish between enamel wear and dentin exposure. Furthermore, Kim et al.^19^ found a strong linear relationship (*r* = 0.994) between fluorescence brightness and stepwise removal depth in extracted teeth, suggesting that QLF is a valuable non-invasive tool for monitoring wear progression and estimating enamel thickness. A clinical study further validated these findings, reporting that higher ΔF_wear_ values correlated with an increased likelihood of dentin exposure, with a proposed threshold of 12–15% (AUROC 0.87–0.93) applicable across different age groups^20^. These findings indicate that QLF-derived parameters can serve as quantitative indicators of cumulative tooth wear.

The diverse wear patterns and corresponding fluorescence parameters across multiple teeth constitute a complex multidimensional dataset. Traditional statistical methods, such as linear regression, are often insufficient to fully capture the intricate interactions and non-linear relationships between these variables and chronological age. To address this challenge, artificial intelligence (AI) has emerged as a powerful alternative capable of learning complex patterns from data to build predictive models. The application of AI techniques with image analysis is an active area of research in dentistry aimed at enhancing diagnostic precision^21^. Numerous studies have successfully applied AI to dental radiographs and photographs to detect caries^22–24^, and periodontal disease^25–27^. This synergy between imaging and AI is also being explored for forensic age estimation, where machine learning (ML) models have shown the potential to significantly reduce prediction errors compared to conventional methods. For example, Abuabara et al.^28^ reported a 44% reduction in mean absolute error (MAE) over the traditional Demirjian’s method^29^ by applying ML models to tooth development stages in panoramic radiographs. Similarly, Dogan et al.^30^ demonstrated that an ML model based on the pulp/tooth area ratio from periapical radiographs could partially account for the non-linear patterns that were missed by linear regression.

While AI techniques excel in learning complex relationships to predict continuous variables such as age, their clinical adoption hinges on explainability and reliability. The “black box” nature of some AI models can create user skepticism, hindering their practical implementation in healthcare^31^. Despite their high performance, deep learning (DL) models have faced scrutiny due to their opaque decision-making processes, which limit their clinical acceptance^32,33^. This has led to a growing emphasis on explainable AI (XAI), which advocates approaches that provide insight into the model’s decision-making basis. Recent literature emphasizes that transparency and explainability are not merely technical preferences but ethical prerequisites for establishing trust and ensuring human oversight in high-stakes medical applications^34^. The random forest (RF) algorithm, an ensemble ML technique, offers a compelling solution. It builds multiple decision trees to deliver a robust performance while mitigating overfitting. Crucially, it provides a degree of interpretability by quantifying the contribution of each input variable through “feature importance” scores. This balance between predictive performance and transparency makes RF particularly suitable for exploratory analyses with limited sample sizes. Owing to these attributes, RF is widely used for prediction tasks involving structured tabular medical data and has demonstrated high accuracy in various clinical applications^28,30^. The quantitative data extracted from biofluorescence images, as in our study, are particularly well-suited for such RF-based ML techniques.

**Fig. 1:**
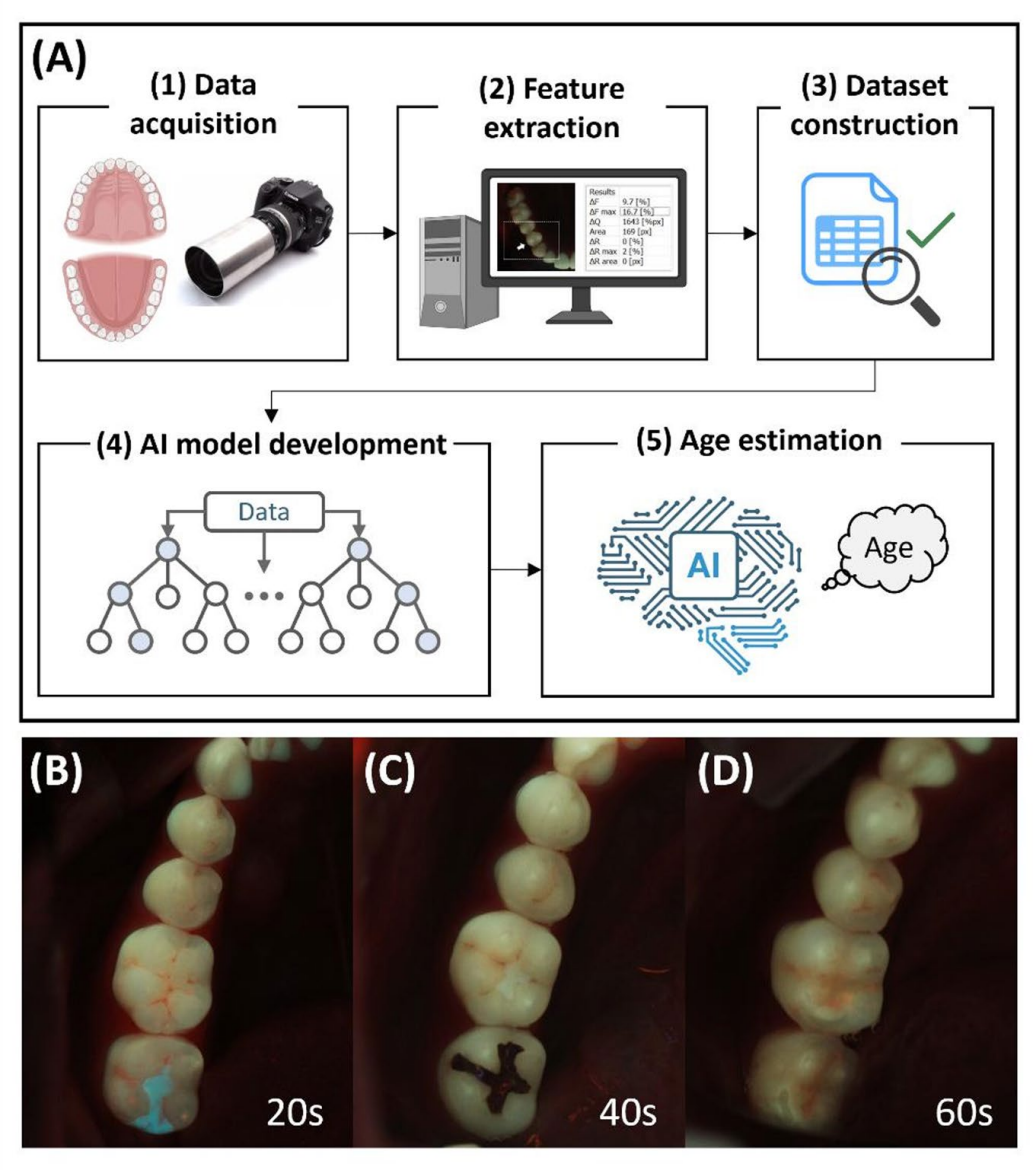
Analytical workflow of AI-assisted age estimation using biofluorescence. (**A**) Workflow schematic with five steps: (1) Data acquisition: chronological age and QLF images were obtained from 104 participants (2,733 teeth). (2) Feature extraction: The ΔF_wear_ values were calculated from the occlusal biofluorescence images. (3) Dataset construction: A participant-level table was built with chronological age and ΔF_wear_ values for each tooth, which was then split into training and test sets. (4) AI model development: Random forest regression was applied, incorporating model training, validation, and RFECV-based feature selection. (5) Age estimation: Chronological age predicted from ΔF_wear_ values using the trained model. (**B–D**) Representative QLF images of participants in their 20s, 40s, and 60s, illustrating age-related differences in fluorescence intensity (bright regions) on the occlusal cusps.

Furthermore, analytical efficiency is a key consideration for the practical use of age estimation models in clinical and forensic settings. Analyzing all 28 teeth (excluding third molars) is time-consuming and may be unfeasible in cases with missing or restored teeth. This issue is analogous to the use of “index teeth” in other dental assessments, such as periodontal examinations^35,36^ and oral hygiene evaluations^37^, where a few representative teeth are examined to infer the status of the entire dentition. Feature importance analysis from RF provides a data-driven tool for identifying index teeth for age estimation. Objectively selecting a small set of key teeth that best represent the overall wear pattern can enhance the clinical utility and generalizability of age prediction models. To provide an overview of this approach, Fig. 1 summarizes the conceptual framework and analytical workflow of the proposed AI-assisted age estimation strategy based on biofluorescence-derived tooth wear features.

Accordingly, this study was designed as a feasibility and proof-of-concept investigation to explore whether a quantitative biofluorescence-derived tooth wear parameter (ΔF_wear_) can be integrated with an interpretable ML framework for adult age estimation. The objectives of this study were as follows: (1) to evaluate the correlation between ΔF_wear_ and chronological age to verify the biological validity of the fluorescence-based wear parameter; (2) to develop and evaluate the predictive performance of an RF regression model; and (3) to identify an optimized subset of key teeth using a recursive feature elimination with cross-validation (RFECV) algorithm, thereby establishing a simplified and clinically applicable model.

## Results

### Participant characteristics and distribution of ΔF_wear_ by age group

A total of 104 participants were categorized into five age groups, ranging from their 20s to 60s. The distribution of participants and median ΔF_wear_ values with interquartile ranges (IQR) for each age group are presented in Table 1. The median ΔF_wear_ value showed a gradual increase with age, and statistically significant differences were observed among the three major age categories: 20–29, 30–49, and 50–69 years of age.

**Table 1:** Participant Distribution and ΔF_wear_ by age group.

Age group (Year)	*n* (%)	ΔF_wear_ (%)	IQR (Q1-Q3)
20–29	25 (24.0)	10.24^a^	8.69–12.65
30–39	26 (25.0)	12.77^b^	11.46–15.39
40–49	22 (21.2)	15.17^b^	14.28–16.05
50–59	20 (19.2)	17.43^c^	16.63–19.99
60–69	11 (10.6)	17.99^c^	17.34–19.39

*n* indicates the number of participants in each age group. ΔF_wear_ values are presented as median and interquartile range (IQR, 25%–75%) due to partial non-normal distribution. Different superscript letters indicate statistically significant differences between groups (*p* < 0.05; Bonferroni-corrected Mann-Whitney *U* test).

### Intra-examiner reliability of ΔF_wear_ quantification

The reliability of ΔF_wear_ quantification was validated by assessing the intra-examiner reproducibility. The re-evaluation of 30 tooth samples, stratified by age group, yielded an intraclass correlation coefficient (ICC) of 0.98 (95% CI: 0.96–0.99).

### Correlation between ΔF_wear_ and chronological age

**Fig. 2:**
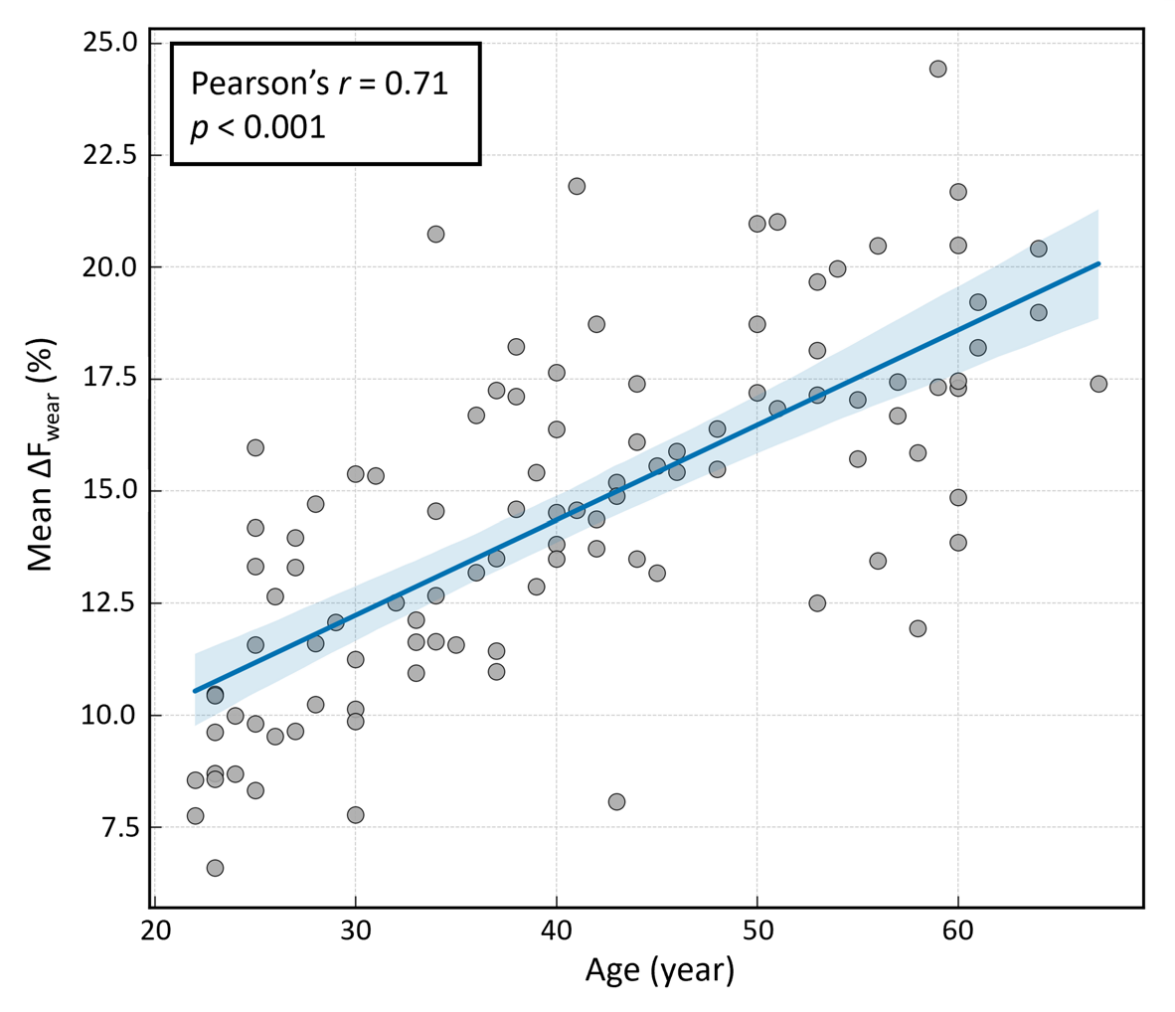
Correlation between chronological age and mean ΔF_wear_ (%) across all teeth. The scatter plot displays the relationships for all 104 participants. The solid line represents the linear regression trend, and the shaded area indicates the 95% confidence interval of the trend. Pearson’s correlation coefficient (*r*) and *p*-value are shown in the top-left corner.

Pearson’s correlation analysis was performed to assess the relationship between chronological age and the mean ΔF_wear_ value (averaged across all 28 teeth) for each participant. A statistically significant positive correlation was confirmed (*r* = 0.71, *p* < 0.001), as illustrated in the scatter plot (Fig. 2).

### AI model optimization and feature selection

Following Bayesian optimization to maximize the predictive performance of the RF model, the optimal hyperparameter combination was determined as follows: n_estimators = 100, max_depth = 12, min_samples_split = 3, and min_samples_leaf = 2. This optimized model demonstrated stable performance, achieving a mean MAE of 7.32 (± 1.22) years in the 5-fold cross-validation (CV) of the training set.

**Fig. 3:**
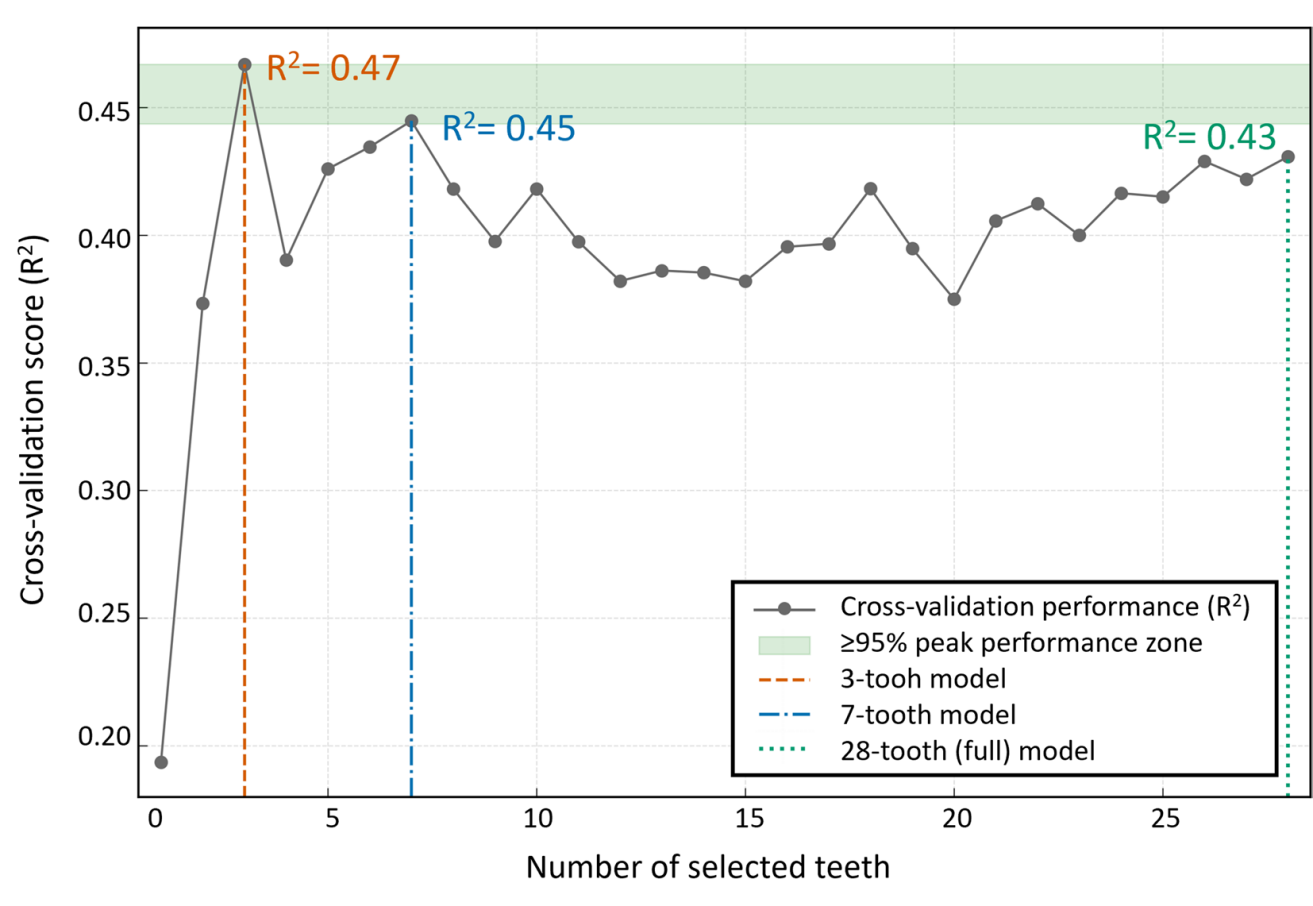
Identification of the optimal number of features using recursive feature elimination with cross-validation (RFECV). The plot shows the mean 5-fold cross-validation performance (R^2^) on the training set as a function of the number of selected features (teeth). The performance peaked with the 3-tooth model (R^2^ = 0.47). A selection criterion was established for models performing at ≥ 95% of peak performance (≥ 95% peak performance zone). The 7-tooth model (R^2^ = 0.45), exhibiting the second-highest performance and satisfying this criterion, was selected as the final candidate while providing a balance between performance and model complexity. The 28-tooth model (R^2^ = 0.43) was included for comparison.

RFECV was then performed using this optimized model to explore the change in CV performance (R^2^) as a function of the number of teeth (Fig. 3). The CV R^2^ peaked at 0.47 with three teeth. The 7-tooth model achieved the second-highest performance (R^2^ = 0.45), which was within 95% of the peak R^2^. Based on these RFECV results, the 3-tooth, 7-tooth, and 28-tooth (full) models were selected as the final candidates for a comprehensive evaluation.

### Prediction performance of the models

In this study, teeth were denoted using a two-digit system from the Fédération Dentaire Internationale (FDI). The 3-tooth combination identified by the recursive feature elimination (RFE) algorithm consisted of teeth 11, 27, and 44; the 7-tooth combination comprised teeth 11, 16, 17, 21, 27, 31, and 44.

The final comprehensive performance comparison of these three candidate models, evaluated using the independent test set, is presented in Table 2. The 7-tooth model achieved an MAE of 7.49 years (95% CI: 5.90–9.17). Its performance did not differ significantly from that of the 28-tooth model (MAE: 7.27 years; *p* = 0.79), while both models showed identical R² values (0.61). Conversely, the 3-tooth model (MAE: 9.11 years) recorded a significantly higher MAE than the other two models (*p* < 0.05), with a lower R² value (0.40). The Pearson’s correlation coefficient between the mean ΔF_wear_ of the selected teeth and age was higher for the 7-tooth model (*r* = 0.78) than for the 28-tooth model (*r* = 0.71). Furthermore, in the training set stability assessment (5-fold CV), the 7-tooth model also showed the most stable performance (mean MAE: 7.03 ± 1.52 years), followed by the 28-tooth model (7.32 ± 1.22 years) and the 3-tooth model (8.24 ± 0.94 years).

**Table 2:** Comprehensive performance comparison of the candidate models.

Model type	Selected teeth (FDI number)	MAE (years)	95% CI	*R* ^2^	ΔF_wear_-Age *r*
3-tooth	11, 27, 44	9.11^a^	7.23–11.51	0.40	0.69
7-tooth	11, 16, 17, 21, 27, 31, 44	7.49^b^	5.90–9.17	0.61	0.78
28-tooth	All teeth	7.27^b^	5.46–9.25	0.61	0.71

Different superscript letters indicate statistically significant differences between groups (*p* < 0.05; Wilcoxon signed-rank test). MAE: mean absolute error; CI: confidence interval; R^2^: coefficient of determination, ΔF_wear_ -Age *r*: Pearson’s correlation between mean ΔF_wear_ and chronological age.

### Comprehensive analysis of the final selected model

**Fig. 4:**
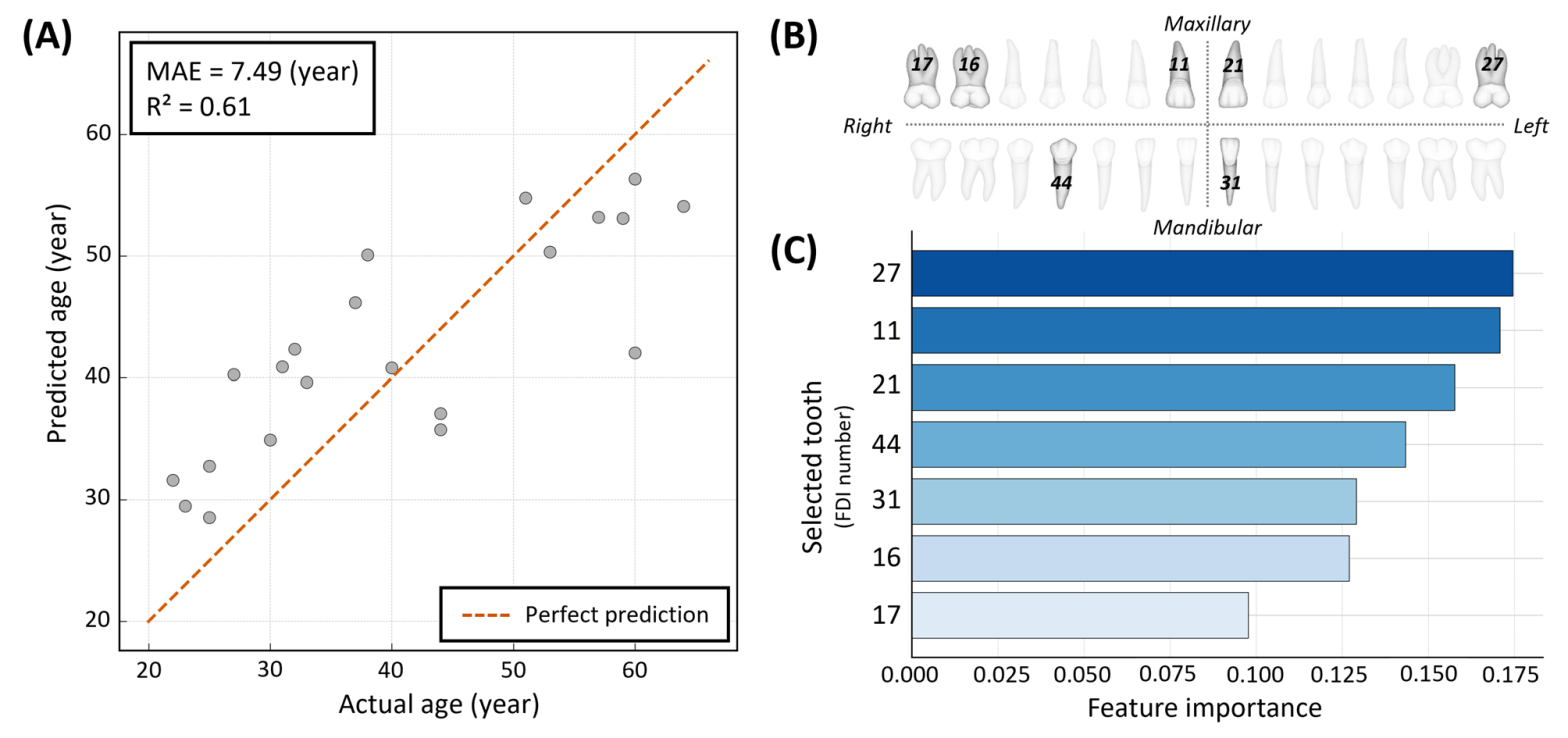
Comprehensive analysis of the final simplified model using 7 key teeth. (**A**) A scatter plot comparing the model’s predicted age with the actual age. The plot is based on an independent test set (*n* = 21). The dashed line represents the ideal line of perfect agreement (y = x). (**B**) Schematic illustrating the anatomical location of the seven selected teeth (11, 16, 17, 21, 27, 31, and 44) based on the FDI notation system. (**C**) A bar plot showing the feature importance within the 7-tooth model, indicating the contribution of each selected tooth to the final prediction.

Based on the comparative performance evaluation, the 7-tooth model was adopted as the final model for this study. Figure 4 presents a comprehensive analysis of this model, integrating its predictive performance (Fig. 4A), and the anatomical locations of the selected teeth (features) (Fig. 4B), and their relative feature importance (Fig. 4C). The feature importance analysis (Fig. 4C) revealed that the maxillary left second molar (27), maxillary central incisors (11, 21), and mandibular right first premolar (44) were the top contributors to age prediction. The final 7-tooth set includes incisors, premolars, and molars distributed across the left and right sides.

## Discussion

This study represents a proof-of-concept investigation suggesting that the occlusal biofluorescence parameter (ΔF_wear_) derived from QLF imaging reflects age-related biological changes. By integrating this parameter with ML, the present work explores the feasibility of a quantitative and non-invasive approach for biological age estimation. In particular, the findings indicate that a simplified subset of teeth may retain age-related information comparable to that obtained from full-dentition assessment, supporting the potential clinical efficiency of the proposed workflow.

The significant correlation between ΔF_wear_ and chronological age quantitatively supports the long-standing hypothesis that cumulative tooth wear reflects biological aging. This finding suggests that ΔF_wear_ derived from dental biofluorescence can serve as a useful indicator of cumulative enamel loss in a population. The median ΔF_wear_ values also increased progressively across age groups, reflecting the continuous nature of wear. However, Lucas et al.^38^ reported that mammalian tooth wear generally increases with age but may vary widely between individuals depending on diet and occlusal patterns, introducing substantial inter-individual variability. These uncontrolled factors may have contributed to the variance and residual errors observed in this study. Nevertheless, a correlation coefficient exceeding 0.70 indicates that ΔF_wear_ has strong potential as a biomarker for age estimation.

Using the optimized RF model, age prediction based on ΔF_wear_ demonstrated prediction errors within the range (± 4.2–10 years) reported for existing forensic age estimation methods^7,9^. Conventional forensic age estimation methods vary depending on the biological substrate and assessment strategy. In addition, these techniques require either extracted teeth or radiographic examinations, involving destructive procedures or ionizing radiation exposure, respectively^10^. Alternatives based on visual assessment of occlusal wear have also been proposed, but their performance is inherently influenced by subjective grading and examiner calibration^39,40^. In contrast, the present QLF-based AI approach relies on quantitative image-derived parameters, thereby providing a more standardized basis for age estimation compared with visually graded approaches. Considering the generally accepted forensic tolerance threshold of ± 10 years proposed by Solheim and Sundnes^41^, the observed prediction error suggests that this approach may serve as a viable preliminary screening tool.

To enhance the model efficiency while maintaining the predictive performance, a systematic feature selection procedure based on RFECV was implemented. This algorithm objectively explores the performance of all possible feature subsets according to the CV scores, minimizing the subjective bias in model construction. The observation that the model performance peaked when using three and seven features led to the selection of a 7-tooth combination using the RFE algorithm. This data-driven approach ensured that the final model was optimized empirically rather than by arbitrary selection. Notably, the selected combination included both anterior and posterior teeth, reflecting the functionally distinct aspects of tooth wear (Fig. 4B). The anterior teeth primarily represent localized wear due to incisal function and parafunctional habits, whereas the posterior teeth mainly reflect cumulative occlusal wear from mastication^42^. Therefore, the combination of anterior and posterior teeth likely provided complementary information that contributed to the predictive strength of the model.

Previous studies have attempted to estimate age using selected representative teeth rather than the entire dentition, suggesting that reduced tooth sets may provide practical alternatives to full-mouth assessment^40,43,44^. However, approaches based on visual grading or highly simplified tooth subsets have shown variable accuracy and may suffer from reduced robustness when excessive simplification is applied^44^. Conversely, the present study utilized a data-driven AI-based feature-selection algorithm to objectively determine the optimal combination of seven key teeth. This optimized model achieved explanatory performance comparable to that of the 28-tooth model while reducing the number of variables by approximately 75%. The stronger correlation with age exhibited by the seven selected teeth compared to the full dentition suggests that the inclusion of the full dentition likely introduced “noise” from teeth with weak age correlations. The feature-selection process likely filtered out these non-informative variables, thereby enhancing the signal-to-noise ratio.

An interesting finding emerged when comparing the CV results with those from the independent test set. While the 3-tooth model appeared optimal during CV, its performance was less stable in the independent test evaluation. In contrast, the 7-tooth model demonstrated more consistent performance across validation strategies, highlighting the importance of independent validation beyond in-sample performance. The final 7-tooth model included a balanced distribution of anterior and posterior, maxillary and mandibular teeth, enabling robust performance even in cases with localized tooth loss or restorative materials. In situations where certain teeth cannot be analyzed, k-nearest neighbors (KNN) imputation or feature-importance-based substitution models can be considered. Therefore, the proposed 7-tooth model may serve as a simplified index that allows clinicians and forensic experts to perform age estimation more rapidly and consistently in practical settings.

The integration of biofluorescence technology and AI could be applicable across a range of clinical and forensic contexts. In contrast to conventional approaches that require destructive procedures or rely on subjective visual assessment^10,30,44,45^, QLF provides a non-invasive approach that minimizes procedural complexity and facilitates data acquisition under constrained conditions. These attributes make it particularly suitable for clinical settings, including vulnerable populations, as well as for diverse forensic applications. Furthermore, large-scale epidemiological studies could be conducted using only QLF devices and analysis software, and the mean ΔF_wear_ values may serve as indicators for population-level assessments of age distribution, nutritional status, and oral health conditions. With remote image analysis systems, this technology could also support tele-dentistry-based age estimation in regions with limited access to dental professionals^20^.

The RF algorithm offers model interpretability by quantifying the relative importance of each feature, allowing clinicians and forensic experts to understand and trust the model’s decision-making process. Compared with complex deep learning models that require substantial computational resources, RF provides a more practical and transparent solution, an advantage that aligns well with the growing emphasis on XAI in modern healthcare. This focus on interpretability is consistent with recent ethical frameworks advocating for AI systems designed to augment human expertise rather than acting as autonomous replacements, ensuring decision-making remains verifiable and explainable^46^. In this study, the model identified specific teeth whose wear patterns were influential for age estimation, contributing to improved interpretability and potential clinical acceptability of AI-assisted analysis. To further enhance transparency and facilitate practical application, visualization techniques such as Shapley Additive Explanations (SHAP), a widely used framework in explainable AI, can be employed to illustrate the contribution of each feature to individual predictions, enabling AI to function not merely as a predictive tool but as a complementary aid to expert judgment.

This study has several limitations that need to be acknowledged and suggest directions for future research. First, as a cross-sectional study, it confirmed the correlation between chronological age and ΔF_wear_ but could not capture longitudinal changes in individual tooth wear over time. Second, this study was based on a relatively small sample of 104 participants from a single institution, with a limited representation of individuals aged over 60 years. The resulting imbalance in age distribution may have influenced model performance in older age groups and restricted age-specific interpretation of the results. Although the analysis was performed at the tooth level (*n* = 2,733), allowing for an exploratory ML-based assessment of tooth-level wear patterns, the findings should be interpreted with caution, as the generalizability at the individual level remains limited. Considering that QLF technology is not yet widely adopted in routine clinical practice compared to conventional modalities, large-scale data acquisition remains challenging. In this context, the present study provides initial empirical evidence supporting the feasibility of applying AI-based analysis to QLF-derived biofluorescence imaging. Future studies with larger and multi-institutional datasets are needed to enhance external validity and generalizability. Third, although pathological factors of tooth wear (e.g., bruxism, erosion, and severe oral habits) were excluded during the study design, it remains difficult to fully control for subtle lifestyle-related variables. Subsequent research should include individuals with pathological wear patterns as a separate group to compare the discrepancy between chronological and ΔF_wear_-based estimated ages, which may provide insight into early detection and quantitative evaluation of pathological tooth wear. Fourth, the quantification of ΔF_wear_ relied on manual ROI identification, which may introduce operator-dependent variability. However, this potential limitation was mitigated by the use of a standardized analysis protocol, and the high intra-examiner reliability observed in this study demonstrated that consistent and reproducible quantification is achievable even with manual contouring. Nevertheless, the semi-automated nature of the current analytical workflow highlights the need for further methodological refinement. Future studies should aim to develop fully automated or AI-assisted ROI detection methods to further enhance objectivity, scalability, and clinical applicability.

Building upon this need for automation, this study holds substantial potential for advancement through more sophisticated AI techniques. While the present model relied on feature extraction from segmented ROIs, future work could employ DL-based approaches to support more automated analysis of raw QLF images and identification of complex wear patterns—including fluorescence intensity, spatial extent, and distribution—for age estimation. Future research should also explore multimodal integration of diverse oral indicators beyond biofluorescence. By combining QLF data with complementary information such as natural tooth color under white light^10^, fluorescence hue^47^, and anatomical features related to the root apex and pulp chamber, an advanced and comprehensive framework for dental age estimation could be established.

The present study suggests that biofluorescence-derived tooth wear features can be incorporated into an explainable machine-learning framework for age-related assessment. This framework is designed to support efficient and interpretable analysis, and may complement existing methods for age estimation. Further validation using larger and more diverse cohorts, including longitudinal data, will be necessary to clarify its generalizability and to refine its applicability across different populations.

## Methods

### Participants and ethical approval

This investigation was a secondary analysis performed on a de-identified dataset collected from a prior study, which was approved by the Institutional Review Board (IRB) of Yonsei University Dental Hospital (Approval No. 2-2015-0030)^20^. Written informed consent was obtained from all participants in the original study. All methods were performed in accordance with the relevant guidelines and regulations, including the Declaration of Helsinki. The current analysis utilized only age and QLF image data, with all personally identifiable information removed. De-identified QLF image data from 104 participants were included in this analysis.

### Acquisition of clinical images using QLF

For each participant, one intraoral image of the maxillary arch and one of the mandibular arch were captured. Images were acquired using a QLF-D Biluminator 2 + system (Inspektor Research Systems BV, Amsterdam, Netherlands) with a 405 nm blue light source. To ensure optimal image quality, all imaging was performed in a darkroom. Two images were obtained for each view: a standard white-light image (shutter speed: 1/30s, Aperture: f/8.0, ISO: 1600) and a corresponding biofluorescence image (shutter speed: 1/30s, Aperture: f/5.0, ISO: 1600). The participants’ lips and soft tissues were retracted, and an occlusal mirror was used to capture the occlusal surfaces. This study retrospectively analyzed QLF images and the corresponding chronological ages.

### Sample selection and inclusion criteria

A total of 104 participants were enrolled in this study. Fluorescence and white-light images of each participant were independently screened for eligibility by two blinded examiners who were calibrated in QLF image analysis and tooth wear assessment. Participants with clinical signs of pathological wear, such as bruxism, severe parafunctional habits, or erosion, were excluded to ensure that the sample reflected physiological wear.

At the tooth level, the following exclusion criteria were applied: (1) missing teeth, (2) presence of restorations on the occlusal cusps, (3) poor image quality or artifacts preventing reliable ΔF_wear_ measurement, (4) partial obstruction of the tooth by the lips or mirror, and (5) third molars. Only teeth unanimously included by both examiners during the screening process were used in the final analysis. Following this screening process, 2,733 teeth were included for ΔF_wear_ quantification.

### Biofluorescence image analysis

**Fig. 5:**
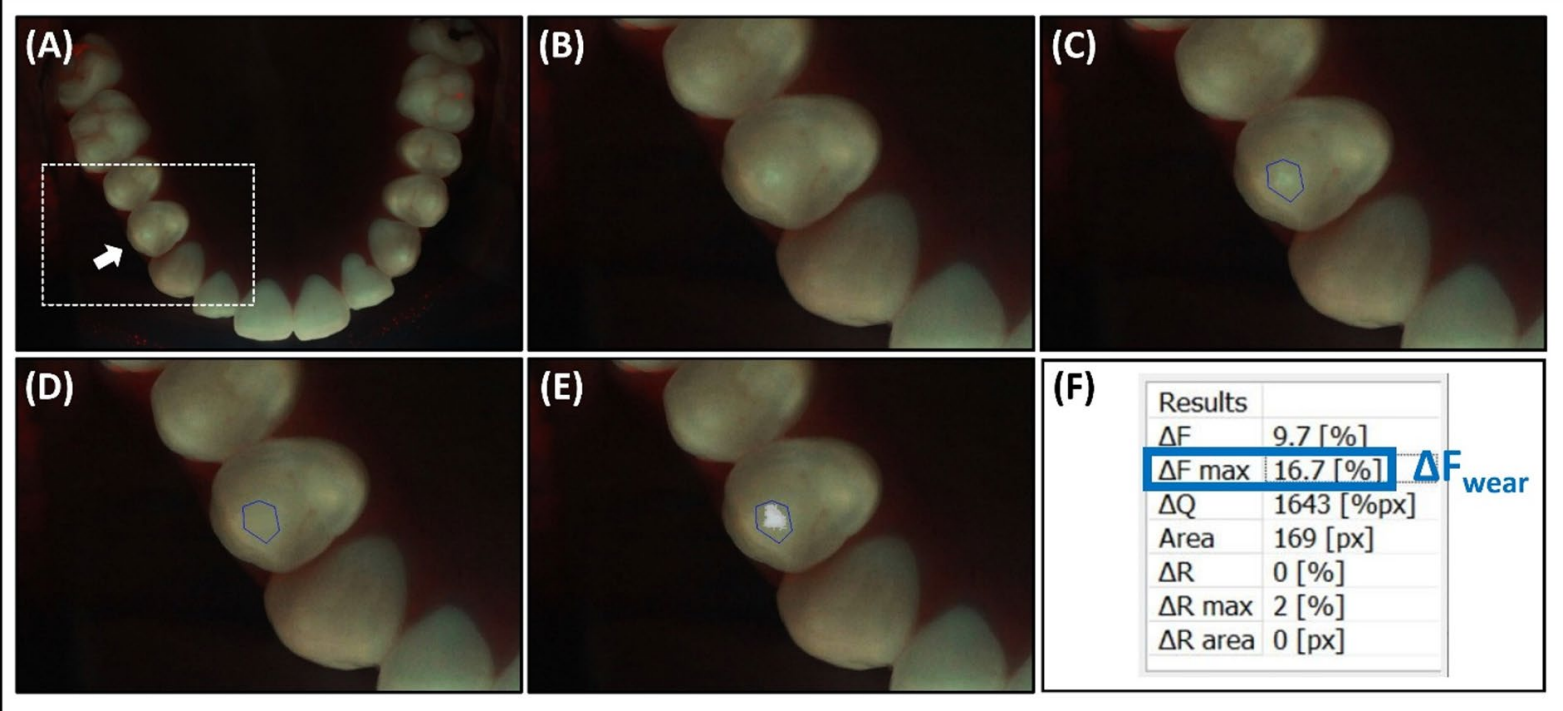
Biofluorescence image analysis procedure for calculating ΔF_wear_ on occlusal surfaces. (**A**) Representative occlusal biofluorescence image captured using QLF. The area selected for analysis is marked with a dashed rectangle and arrows. (**B**) Magnified view of the analysis area. (**C**) Region of interest (ROI) manually contoured to encompass the wear-related high-fluorescence area, with the boundary placed on the surrounding sound enamel while excluding unrelated fluorescence patterns. (**D**) Reconstructed image simulating the fluorescence of sound enamel. (**E**) Fluorescence difference between original and reconstructed images. (**F**) Quantitative results generated by the analysis software.

For each of the 2,733 selected teeth, the change in fluorescence intensity was analyzed in the most severely worn occlusal region. All quantitative analyses, including manual ROI contouring and ΔF_wear_ calculation, were performed by a single trained examiner to ensure analytical consistency. The ΔF_wear_ parameter was quantified using dedicated QLF analysis software (QA2J, version 2.0.0.18, Inspektor Research Systems). This analysis is based on the principle that worn areas (worn enamel or exposed dentin) exhibit a higher fluorescence intensity than the surrounding sound enamel. The region of interest (ROI) was manually contoured to encompass the area of increased fluorescence, with the ROI boundary placed on the surrounding sound enamel to establish reference value (F_sound_), according to the standardized analysis protocol detailed in Fig. 5. Within this ROI, the maximum fluorescence gain (ΔF_max_) was calculated.

The ΔF_wear_ value, representing the percentage difference in fluorescence between the worn site (F_max_) and the reference value (F_sound_), was calculated using Eq. (1):

1 $${\Delta}F_{wear}\%=\left(\frac{{F}_{max}-{F}_{sound}}{{F}_{sound}}\right)\times100$$

Consistent with the protocol, fluorescence patterns unrelated to wear were not included in the ROI, such as areas exhibiting fluorescence loss (e.g., anatomical fissures, carious lesions, or cracks) or regions showing red fluorescence associated with dental plaque or calculus^48,49^. These signals are optically distinct from both sound enamel and wear-related fluorescence. For each tooth, the highest ΔF_wear_ value observed among all cusp areas was selected as the representative value.

### Reliability assessment of ΔF_wear_ quantification

To address potential variability associated with manual ROI identification, an intra-examiner reliability analysis was conducted. A stratified random subset of 30 teeth was selected, comprising six samples from each age group (20s, 30s, 40s, 50s, and 60s), to ensure representation across the full spectrum of wear severity. The corresponding images were re-evaluated by the same examiner, and the reproducibility of ΔF_wear_ quantification was assessed using the intraclass correlation coefficient (ICC).

### Statistical analysis and age prediction model development

All statistical analyses and ML model development were performed using Python (version 3.11), primarily with the *scikit-learn* library (version 1.7.1). The analysis workflow was structured as follows: (1) data preprocessing and splitting, (2) RF model optimization, and (3) a multi-step validation strategy for model selection and performance evaluation.

### Data preprocessing and splitting

To strictly prevent data leakage, the entire dataset of 104 participants was split at the participant level using the GroupShuffleSplit function of *scikit-learn*. The data were partitioned into a training set (80% of participants) and an independent test set (20% of participants). The random seed was fixed (random_state = 42) for reproducibility.

For each participant, the ΔF_wear_ values from all available teeth (up to 28, excluding third molars) were used as independent variables (features), and chronological age was used as the dependent variable (target). The number of features corresponded to the number of teeth in the dentition. All preprocessing steps, including the imputation of missing values (due to excluded teeth) using KNN (k = 5), were integrated into a *scikit-learn* Pipeline. This ensured that imputation was performed only after splitting and within the CV folds, preventing any information from the test set from influencing the training process.

### Random Forest model optimization

To optimize the predictive performance of the model, Bayesian optimization (BayesSearchCV) was applied to the training set to determine the best hyperparameter combination for the RF regression model. Based on common practice, max_features was fixed to the square root. The optimization search space included n_estimators, max_depth, min_samples_split, and min_samples_leaf. This process was guided by 5-fold CV at the participant level (GroupKFold), with the objective of minimizing the MAE.

### Multi-step strategy for model selection and evaluation

A systematic, multi-step strategy was employed to select and comprehensively evaluate the final models used.

First, in the model design phase, RFECV was performed on the training set using the optimized RF model described above. We analyzed the RFECV performance curve, which plots the CV score (R^2^) against the number of features (teeth). Based on this curve, candidate models with a reduced number of key teeth were selected for further analysis. Subsequently, recursive feature elimination (RFE) was used to determine the specific combination of teeth for each candidate model size.

Next, in the independent test set validation, the candidate simplified models from RFE and the full model using all 28 potential teeth were evaluated on the independent 20% test set, which was held out from all training and optimization. The primary performance metrics were MAE with a 95% confidence interval (CI) and the coefficient of determination (R^2^). Additionally, Pearson’s correlation coefficient (*r*) between the mean ΔF_wear_ and chronological age was calculated. A Wilcoxon signed-rank test was conducted for pairwise comparisons of the MAE values between the candidate models to assess the statistical significance of the performance differences.

Finally, for model stability validation, a 5-fold CV was performed within the 80% training set to assess the stability and consistency of each selected candidate model by evaluating the mean MAE and its standard deviation across the folds.

## Data Availability

The datasets and analysis code used and analyzed during the current study are available from the corresponding author on reasonable request.
